# Chemical Variation, Antimicrobial, Nitric Oxide Scavenging Activities and Tyrosinase Inhibition of Essential Oils and Solvent Extracts from *Filipendula vulgaris* Moench Growing in Turkey

**DOI:** 10.22037/ijpr.2021.114302.14786

**Published:** 2021

**Authors:** Seda Fandakli, Büşra Korkmaz, Özlem Faiz, Gözde Kiliç, İshak Erik, Salih Terzioğlu, Nurettin Yaylı

**Affiliations:** a *Department of Diet and Nutrition, Faculty of Health Sciences, Avrasya University, Trabzon, Turkey. *; b *Department of Pharmacognosy, Faculty of Pharmacy, Karadeniz Technical University, Trabzon, Turkey. *; c *Department of Chemistry, Faculty of Arts and Sciences, Recep Tayyip Erdogan University, Rize, Turkey. *; d *Department of Forest Botany, Faculty of Forestry, Karadeniz Technical University, Trabzon, Turkey.*

**Keywords:** Filipendula vulgaris, essential oil, GC-FID/MS, SPME, biological activity.

## Abstract

Volatile organic compositions of the essential oils (EOs), solid-phase microextraction (SPME) and SPME of *n*-hexane extracts from the flower and stem-leaf of *Filipendula vulgaris (F. vulgaris) *were analyzed by GC-FID/MS. A total of 107 constituents were characterized, flower and stem-leaf parts of the plant were found to contain different volatile organic compounds. Tricosane (29.6%), *n*-nonanal (20.5%) were identified as the main components in the essential oil of the flower, while phytol (35.2%) was found to be a major constituent in the essential oil of stem-leaf*. *Benzaldehyde (56.0%) and *n*-nonanal (31.6%) were the major groups in the SPME of stem-leaf and flower, respectively. The volatiles for the SPME of *n*-hexane extracts of the flower and stem-leaf of *F. vulgaris *were predominated by aromatic compounds (75.0% and 78.5%) and ketones (18.1% and 10.1%), respectively. On the other hand, a total of terpene compounds was found at the most in the EO of the stem-leaf part of the plant (48.6%). In addition, antimicrobial, tyrosinase inhibition, and nitric oxide scavenging activities of the *n*-hexane (H), methanol (M), aqueous extracts (A) and EOs of *F. vulgaris *were investigated. EOs and methanol extracts of flower and stem-leaf had high antimicrobial activity against tested various microorganisms*.* However, *n*-hexane extracts of the flower and stem-leaf only displayed activity against* Mycobacterium smegmatis*. Methanol extracts of flower and stem-leaf possessed the best tyrosine inhibition and NO scavenging activity.

## Introduction

The genus *Filipendula* Mill. has 15 taxa throughout the world (1), mainly in the N Temperature zone (2). In Turkey, it is represented by two species in Flora of Turkey, such as *F. vulgaris* Moench and *F. ulmaria* (L.) Maxim. (3, 4). *F. vulgaris *(syn. *Spiraea filipendula *L., and *Filipendula hexapetala *Gilib.), commonly known as dropwort or fern-leaf dropwort, which is a rhizomatous perennial herb of the family Rosaceae. It is found in dry pastures across much of Europe and central and northern Asia, mostly on lime (5). The roots of *F. vulgaris* have been used as a folk medicine to treat kidney diseases, shortness of breath, wheezing respiration, pain in the throat, abdominal pain and diarrhea in European countries (Bulgaria, Poland, Serbia and Ukraine) (6, 7). In addition, tea prepared from leaves of *F. vulgaris* is used to relieve influenza and gout, to clean wounds and eyes (6).

Previous studies on volatile components of *F. vulgaris* as; the EO of the aerial parts of *F. hexapetala* from central Western Serbia (7), the essential oil from the leaf of *F. vulgaris* (6), the GC-​MS-​analysis for the water-alcohol extracts of the root, flower, and leaf extracts of *F. hexapetala* (8) and SPME GC-MS analysis of Filipendula (meadowsweet) genus (*F. angustiloba *Maxim*., F. camtschatica *Maxim*., F. denudata *Fritsch*., F. glaberrime *Nakai*, F. intermedia *Juz*, F. palmata *Maxim*., F. picbaueri *Smejkal.*, F. ulmaria *Maxim*, *and* F. vulgaris* growing on Eurasia) were mentioned (9). In the literature, antimicrobial, anti-inflammatory, genotoxicity, analgesic, and antioxidant activities of the EOs and solvent extracts (methanolic, ethanolic, aqueous, and aqueous ethanolic extracts) of *F. vulgaris* had studied (10-18). And also, phytochemical studies in *F. vulgaris *revealed the ascorbic acid, hydrolyzable tannins, flavonoids, procyanidins, phenol carboxylic acids, salicylic acid, polysaccharides, traces of coumarone and other derivatives as natural compounds (19-29). 

The volatile organic content from the EOs, SPME and SPME of *n*-hexane extracts in the different parts (flower and stem-leaf) of *F.* *vulgaris* grown in Turkey has not been studied previously. In this study, we aimed to investigate the volatile organic composition and biological activities for the parts of *F.* *vulgaris*. In the present work, the volatile content of the EOs, SPME and SPME of *n*-hexane extracts obtained from flower and stem-leaf of *F. vulgaris* were analyzed by GC-FID/MS. In addition, tyrosinase inhibition, nitric oxide scavenging activity and antimicrobial effects of *n*-hexane, methanol, aqueous extracts and EOs of flower and stem-leaf were evaluated.

## Experimental

*Plant materials*

Wild grown *F.* *vulgaris* was collected from 1650 m above sea level in July 2018 from Koyulhisar-Sivas, which is in the northeast part of Turkey. Flower and stem-leaf were air-dried in the shade at room temperature and analyzed as soon as possible. The plant was authenticated by Prof. S. Terzioglu (1, 3-5). The voucher specimen was deposited in the Herbarium of Karadeniz Technical University, Faculty of Forestry (KATO: 16001), Turkey.

*Hydrodistillation (HD) procedure for the isolation of EO*

Flower and stem-leaf of *F.* *vulgaris* were air-dried then ground into small pieces. Seventy grams of each dried grounded flower and stem-leaf were used to obtain essential oil by hydrodistillation (HD) using a modified Clevenger-type apparatus with a cooling bath (-15 ^o^C) system (3 h) (yield (w/w): 18.1 mg, and 13.4 mg, respectively). The obtained oil was extracted with *n*-hexane (0.5 mL) and dried over anhydrous Na_2_SO_4_ and kept in sterilized dark glass bottles in the refrigerator at 4 ^o^C prior to the analysis.

*n-Hexane, methanol and aqueous extracts obtained from flower and stem-leaf of F. vulgaris*

Grounded parts (flower and stem-leaf) of *F.* *vulgaris *(10 g each) were put into six different flasks (50 mL) and extracted three times with an analytical grade *n*-hexane, methanol and water solvents (15 mL × 3; 6 h each). After the suction filtration, the same extracts were combined and evaporated at the 40 ^o^C to give crude *n*-hexane (0.0896 g and 0.0696 g) and methanol (0.2340 g and 0.2410 g) extracts, respectively. The water extracts obtained from flower and stem-leaf of *F.* *vulgaris* were lyophilized to give crude water extracts (0.2340 g and 0.2410 g), respectively (30).

*Solid-phase microextraction (SPME) analysis*

The blended parts (flower and stem-leaf) of dried plant (1.2 g each), and *n*-hexane (30.9 mg and 29.2 mg) extracts (0.1450) of *F.* *vulgaris *were placed to a sealed SPME vial (10 mL) with a silicone-rubber septum cap then submitted to solid-phase microextraction device (Supelco, USA). A DVB/Carboxen/PDMS coating fiber was used to obtain volatile components. The SPME fibers were conditioned for 5 min at 250 °C in the GC injector. Extraction was achieved with magnetic stirring at 80 °C using an incubation time of 5 min and an extraction time of 10 min. Fiber with extract of volatile compounds was subsequently injected into the GC injector. GC-FID/MS analyzes were performed using a Shimadzu QP2010 Ultra mass selective detector attached to the 2010 Plus chromatograph. The carrier gas used was helium at a flow rate of 1 mL/min. The injection was performed in split mode (1:30) at 230 ^o^C. The sample was analyzed and reported. The temperature, incubation and extraction time were set according to the reported experiment (31). 

*Gas chromatography-Mass spectrometry (GC-FID/ MS)*

EOs analysis was carried out using a Shimadzu QP2010 ultra GC-FID/MS, Shimadzu 2010 plus FID, fitted with a PAL AOC-5000 plus autosampler and Shimadzu Class-5000 Chromatography Workstation software. The separation was analyzed by means of a Restek Rxi-5MS capillary column (30 mm × 0.25 mm × 0.25 μm) (USA). Essential oil injections to GC-FID/MS was performed in split mode (1:30) at 230 ^o^C. The essential oil solution (1 μL) in *n*-hexane (HPLC grade) was injected and analyzed with the column held initially at 60 ºC for 2 min and then increased to 240 ºC with a 3 ºC/min heating ramp. The oven program was as follows: the initial temperature was 60 ^o^C for 2 min, which was increased to 240 ^o^C at 3 min, the final temperature of 250 ^o^C was held for 4 min. Helium (99.999%) was used as carrier gas with a constant flow rate of 1 mL/min. Detection was implemented in electronic impact mode (EI); ionization voltage was fixed at 70 eV, scan mode (40-450 m/z) was used for mass acquisition. Each sample was analyzed, and the mean was reported.

*Solid Phase Micro Extraction (SPME) analysis*

Volatile components were extracted using the solid-phase microextraction (SPME) technique. SPME is sensitive, easy and speedy for the extraction of analytes from solid samples. 1.0 g of air-dried grounded plant materials and 0.0250 g *n*-hexane extracts were placed in separate vials (10 mL), which were packed with a silicone-rubber septum cap. Volatile components were absorbed onto a polydimethylsiloxane/divinylbenzene fiber. SPME methodology consisted of 5 min incubation at 50 ^o^C for and 10 min extraction. Then, the fibers with extracted volatile components were loaded into the GC-MS injector in split mode. The oven program was the same as the GC-MS condition (32-33). 

*Identification of Volatile Constituents*

Retention indices and chromatographic peaks were used to identify the VOCs. Retention indices were compared with respect to C_6_-C_30_ alkane standards. Individual chromatographic peaks in the mass spectra were compared with the mass spectra of the commercial libraries (FFNSC1.2, W9N11 and NIST) (33-37).

*Antimicrobial Activity Assessment (Agar-well Diffusion Method)*

All test microorganisms which were obtained from the Hifzissihha Institute of Refik Saydam (Ankara, Turkey) were as *Bacillus cereus, *709 ROMA,* Candida albicans *ATCC 60193, *Enterococcus faecalis *ATCC 29212, *Escherichia coli *ATCC 25922, *Mycobacterium smegmatis *ATCC607, *Pseudomonas aeruginosa *ATCC 27853, *Saccharomyces* *cerevisiae *RSKK 251, *Staphylococcus aureus *ATCC 25923 and *Yersinia pseudotuberculosis* ATCC 911. The plant extracts were dissolved in *n*-hexane and methanol to prepare extracts stock solution. Antimicrobial susceptibility of the EOs, *n*-hexanes, methanol and aqueous extracts of *F. vulgaris *were screened using the agar well diffusion method (38-39). Each bacterium and the yeast were cultured in Mueller Hinton (MH) (Difco, Detroit, MI) broth and yeast extract broth, respectively. Then the microorganisms were diluted approximately 106 colony-forming unit (cfu) per mL. For yeast-like fungi, Sabouraud Dextrose Agar (SDA) (Difco, Detriot, MI) was used. Microorganisms were “flood-inoculated” onto MH and SD agars and dried under aseptic conditions. 50 µl of essential oils, *n*-hexanes, methanol and aqueous extracts of *F. vulgaris* were delivered into wells (diameter = 5 mm) opened on agar plates and incubated at 35 °C for 18 h. The *Mycobacterium smegmatis *was grown for 3 to 5 days on MHA plates at 35 °C. Microbial activity was evaluated by measuring the zone diameters. Antimicrobial agents such as Ampicillin (10 μg/mL), streptomycin (10 µg/mL) and fluconazole (5 µg/mL) were used as a positive control. All tests were carried out in triplicates.

*The total phenolic content*

The total phenolic content of extracts (methanol and aqueous) obtained from the flower and stem-leaves parts of F. vulgaris was studied using Folin–Ciocalteu method and the total flavonoid content method respectively. All extracts (methanol and aqueous) (2 µL, 1 mg/mL) were diluted to 3.0 mL with distilled water and 5 μL of Folin–Ciocalteu was mixed with the extract solutions for 3 min. Followed by the addition of 20 µL of sodium carbonate (20% (w/v)). After the incubation of the mixture for 60 min in the dark, their absorbance was measured at 650 nm (30). The total phenolic content was calculated from a calibration curve obtained by using gallic acid as a standard. The total phenolic content was expressed as mg of gallic acid equivalent per g dry weight.

*Nitric oxide scavenging activity assay*

The *n*-hexane, methanol, and aqueous extracts for the flower and stem-leaf parts of *F. vulgaris* were investigated for their nitric oxide (NO) scavenging potential by the Griess reagent method 40). One milliliter of extracts containing different concentrations of them and sodium nitroprusside in phosphate-buffered saline (pH 7.4) at 5 mM final concentration was incubated at 25 °C for 150 minutes. Following the incubation period 0.5 mL of freshly prepared Griess reagent (1% (w/v) sulphanilamide, 0.1% (w/v) naphthylethylenediamine dihydrochloride and 2.5% (w/v) phosphoric acid) was added and the optical density (OD) was measured at 540 nm. NO scavenging capacity was assessed by comparing the OD values of control and extracts containing reaction mixture. NO% scavenging capacity of extracts and gallic acid were calculated using the formula below:

scavenged nitrite oxide% = 

[(OD_control _- OD_sample_)/OD_control_] × 100

*Tyrosinase inhibition assay*

The *n*-hexane, methanol, and aqueous extracts obtained from the flower and stem-leaf of *F. vulgaris* were investigated for their tyrosinase inhibition potentials. A reaction mixture containing 80 μL of Na-phosphate buffer (50 mM, pH 6.8), 5 μL mushroom tyrosinase (2500 U mL^-1^) (**T3824 SIGMA** Tyrosinase from mushroom), and 15 μL of parts of the plant extracts at different concentrations were prepared and pre-incubated at room temperature for ten minutes. After pre-incubation, 30 μL of 5 mM L-DOPA (3,4-dihydroxy-*L*-phenylalanine) solution was added, mixed and incubated for 30 minutes at room temperature. The OD of the formed dopachrome was read at 470 nm with references at 700 nm (Shimadzu UV-1600 spectrophotometer). Kojic acid was used as a positive control. The extract concentration giving 50% (IC_50_) of the original tyrosinase activity was determined (41).

## Results and Discussion

*Chemical composition of the EOs, SPME and SPME of n-hexane extracts*

GC-FID/MS analysis of EOs, SPME and SPME of *n*-hexane extracts for the flower and stem-leaf of* F. vulgaris* revealed a total of 33/44, 19/17 and 33/36 volatile compounds, representing 99.9%/98.7%, 97.4%/99.8%, and 99.9%/99.8%, respectively. The volatile organic compounds of the EOs, SPME and SPME of *n*-hexane extracts for the flower and stem-leave of* F. vulgaris*, their retention indices and percentages are listed in Table 1. Volatile compounds have been listed in the order of elution on the Restek Rxi-5MS column used (33), which were identified by comparison of the registered mass spectrum libraries (NIST, Wiley7NL, FFNSC1.2, and W9N11) and by using the Kovats index (32-36).

Tricosane (29.6%) was found to be the main constituent in the flower EO, while phytol (35.2%) was the major compound in the stem-leaf EO. *n*-Nonanal (31.6%) and benzaldehyde (56.0%) for the SPME and 1-ethyl-3-methylbenzene (26.0% and 19.9%) for the SPME of *n*-hexane extracts were the main components that were identified from flower and stem-leaf of* F. vulgaris, *respectively (Table 1). The different numbers and types of volatile organic compounds were characterized due to the use of three different extraction techniques. By using three different extraction methods, which were described in the material and methods section, a total of 107 different volatile compounds were identified from the GC-FID/MS analysis and their chemical class distribution (monoterpene and sesquiterpene hydrocarbons, oxygenated monoterpenes, sesquiterpenes, aldehydes, aliphatic and aromatic hydrocarbons, esters, ketones, terpene related compounds and others) were given in Table 1.

Aldehydes (68.8% and 86.6%) were the most abundant components in the SPME of flower and stem-leaf of* F. vulgaris*, respectively. Aromatic hydrocarbons (75.0% and 78.5%) were found in high percentages in the SPME analysis of *n*-hexane extract for both parts, respectively. Diterpenoid (35.2 %) was determined as the main component in the EO of stem-leaf, while aldehydes (49.5%) were found to be a major constituent in the EO of the flower (Table 1).

In the literature, the volatile organic compounds of Filipendula (meadowsweet) genus (*F angustiloba *Maxim.*, F camtschatica *Maxim*., F denudata *Fritsch.*, F glaberrime *Nakai*, F intermedia *Juz*, F palmata *Maxim.*, F picbaueri *Smejkal.*, F ulmaria *Maxim*, F vulgaris* Mocnch growing on Eurasia) were searched from the aerial parts of these plants by the method of a SPME GC-MS, and  19 compounds of the phenolic and isoprene structure were reported and salicylaldehyde (70.8%) was the major compound in the SPME of *F. vulgaris*  (9). The leaf essential oil of *F. vulgaris *had been analyzed by GC-MS and consisting mainly of salicylaldehyde (68.6​%) (6). The EO from the aerial parts *F. hexapetala* had been analyzed, and 31 components were characterized. Salicylaldehyde (13.7%) and *n*-nonanal (11.9%) were the major compounds in the EO of the aerial part *F. hexapetala* (7). The GC-​MS-​analysis for the water-alcohol extracts of root, flower, and leaf extracts of *F. hexapetala* has been reported.  Based on resulted data, flowers and leaves were recommended as the best source of medicinal raw material (8).

When the study was compared with the literature, similar compounds were found at different rates. However, more volatile components were characterized in this work. In addition, phytol was detected in 35.2% only in the stem-leaf volatile component of the plant. Moreover, terpenic compounds were mostly found in the stem-leaf part of the plant. In the essential oil and SPME analyses of flower and stem-leaf, aldehyde compounds were seen as the main component. This plant can be used as a source for obtaining aldehyde compounds, and they might be used as a taxonomical marker for the future classification of the *F. vulgaris*. The variations in the volatile organic compounds on parts of *F. vulgaris *may be due to environmental, storage, and analysis conditions. Thus, it could be pointed out that the qualitative and quantitative results of this study were quite different from the previous reports.

*Antimicrobial activity*

The antimicrobial properties of the EOs, *n*-hexanes, methanol and aqueous extracts of *F. vulgaris *were tested by an *in-vitro *agar-well diffusion method (38-39) using *Bacillus cereus (B. cereus), Candida albicans (C. albicans)*, *Enterococcus faecalis*, *Escherichia coli (E. coli),* *Mycobacterium smegmatis (M. smegmatis), Pseudomonas aeruginosa (P. aeruoginosa), Saccharomyces cerevisiae (S. cerevisiae), Staphylococcus aureus (S. aureus) *and* Yersinia pseudotuberculosis (Y. pseudotuberculosis).* Zone diameters were measured in mm, and a decrease in zone diameters indicates the existence of antimicrobial activity. The *n*-hexane extracts of *F. vulgaris *flowers and stem-leaf did not show any antimicrobial activity against studied bacteria except for *M. smegmatis* (Table 2). Both flower and stem-leaf EOs and methanol extracts showed inhibition in zone diameters against studied microorganisms. EOs extracts were more active for gram-positive bacteria, while methanol extracts were more active for gram-negative. *M. smegmatis* is commonly used as a model organism for tuberculosis and leprosy. All extracts except aqueous had an inhibition effect on *M. smegmatis*. These antimicrobial activities indicate the presence of active components in these extracts.

In the previous antimicrobial evaluation of the plant, the leaf essential oil of *F.vulgaris* was screened by the disk diffusion and microdilution broth assays.  The essential oil remarkably inhibited the growth of all of the tested bacteria and fungi (6). Methanolic extracts obtained from the aerial part and root of *F. vulgaris* were evaluated *in-vitro,* and *in-vivo* *anti*-inflammatory effects, as well as their potential cytotoxicity, were assessed.  The extracts demonstrated prominent *in-vivo* *anti*-inflammatory potential upon oral administration in rats.  Especially aerial ex-tract at 100 and 200 mg​/kg significantly inhibited carrageenan-induced edema formation.  From the mentioned report, it can be concluded that *F. vulgaris* extracts possess *anti*-inflammatory properties (19). Methanolic extracts of *F.* *hexapetala* aerial parts and roots exhibited antimicrobial activity against most of the tested bacterial and fungal species (18).  

In the literature, various works mentioned the antioxidant activities for the solvent extracts of *F. vulgaris* as; *Filipendula* extracts possessed strong antioxidant activity comparable with that of a used standard (11). The ethanolic extracts of *F. vulgaris* collected from the West part of Romania showed antioxidant capacity (12). Antioxidant, anti-inflammatory and gastroprotective effects of *F. vulgaris* lyophilized flower infusions reported (13). Antioxidant activity of aqueous and aqueous-​ethanolic (40​%, 70​%, 95​%) extracts of the above-ground parts of *F. vulgaris* were mentioned (14).  Antioxidant potential in methanol, acetone and water extracts of *F. vulgaris* was evaluated by DPPH and ABTS scavenging assays. Methanol and acetone extract is reported to be stronger antioxidants (15).  The antioxidant activity of the methanolic extracts of *F.* *hexapetala* aerial parts and roots and their potential in different model systems.  The results had shown that *F. hexapetala* extracts had considerable antioxidant activity *in-vitro *and great stability in different conditions (18). Pharmacological studies of *F. hexapetala* were reported (16). The flowers of *F.* *hexapetala* had proven the presence of analgesic activity (17). Due to the abundance of antioxidant studies of *F. vulgaris* extracts in literature (11-18), antioxidant studies of extracts have not been conducted.

*Total phenolic content*

The solvent used for the extraction affects the phenolic content of the extracts (30). Methanol and aqueous extracts of *F. vulgaris *exhibited different phenolic content (Table 3). The total phenolic content in methanol extract obtained from flower and stem-leaf of *F. vulgaris *were 230.6 ± 12.1 mg and 110.8 ± 8.6 mg gallic acid equivalents/g, respectively. In the literature, total phenolic content for the methanol and aqueous extracts of the whole plant without a flower of *F. vulgaris *from Lithuania was reported as 346.6 ± 2.1 and 131.9 ± 4.2 mg gallic acid equivalents/g (30). Total phenolic composition depends on the solvent that used for the extraction. Generally, methanol extracts display higher total phenolic content.

*Nitric oxide scavenging activity assay*

Antioxidants rich natural products could be great antioxidants sources against oxidative stress-associated diseases. Because of surplus NO deleterious effects on the cell, the NO concentration must be regulated. *In-vitro* substances having a regulatory effect on NO concentration were studied using sodium nitroprusside as a NO donor (340). Under aerobic conditions, NO is converted to nitrite ions which can be detected with Griess reagent. The *n*-hexane, methanol and aqueous extracts obtained from flower and stem-leaf parts of *F. vulgaris* were examined for their potential NO regulator activity (Table 4). Aqueous extract obtained from flower and stem-leaves of *F. vulgaris* had the lowest effect on NO (IC_50 _= 350.52 ± 5.85 μg/mL and 286.74 ± 6.23 μg/mL, respectively). Methanol extract obtained from stem-leaf of *F. vulgaris* revealed the best NO scavenging activity (IC_50 _= 10.58 ± 1.66 μg/mL), whereas IC_50_ value of ascorbic acid exhibited 5.55 ± 0.21 μg/mL. A positive correlation was determined between the potency of nitric oxide scavenging capacity and the total phenolic content of *F. vulgaris* extracts. Polyphenols are the main compounds that give plants antioxidant properties.

*Tyrosinase inhibition assay*

Tyrosinase inhibitors are used in cosmetic industries for whitening the human skin by reducing melanization. Tyrosinase inhibition potential of methanol and aqueous extracts obtained from flower and stem-leaf parts of *F. vulgaris* was investigated (41-42). The *n*-hexane extract could not be studied because this solvent inhibited tyrosinase itself. *F. vulgaris* extracts IC_50_ values for tyrosinase inhibition varied from 0.28 ± 0.02 μg/mL to 6.05 ± 0.05 μg/mL (Table 5). Methanol extracts obtained from flower and stem-leaf parts of *F. vulgaris* were better inhibitors than aqueous extracts (higher IC_50_ values). Many plant extracts have been reported to have tyrosinase inhibition potential (41-42). The IC_50_ of *F. vulgaris* extracts was lower than reported for many plant extracts (42). A positive correlation between the tyrosinase inhibition potential and the total phenolic content of *F. vulgaris* extracts was also observed. The presence of total phenolic contents in plant extracts is given against oxidative damage.

**Table 1:** GC-FID/MS analysis of the EOs, SPME and SPME of *n*-hexane extracts obtained from flower and stem-leaf of *F. vulgaris*

**No**	**Compounds**	RI*	**RI** ^a^	**%** ^b^					
				**A1**	**A2**	**A3**	**B1**	**B2**	**B3**
	2-Ethyl furan	728	733	**-**	**-**	**-**	5.2	**-**	**-**
	Toluene	782	781	1.3	-	5.6	2.6	**-**	1.8
	Octane	800	802	3.2	-	0.2	-	**-**	0.1
	Capronaldeyhde	803	803	-	2.6	-	2.6	3.5	-
	Butyl acetate	814	814	-	-	0.6	-	**-**	0.3
	Furfural	836	836	0.4	2.9	-	-	**-**	-
	(*E*)-2-Hexenal	852	852	0.8	2.3	-	1.7	10.1	-
	(*Z*)-2-Hexanol	865	865	**-**	-	-	-	3.1	-
	4-Methyl octane	868	868	**-**	-	0.1	-	**-**	-
	Ethylbenzene	871	871	**-**	-	1.4	-	**-**	0.7
	*p*-Xylene	878	878	**-**	-	7.4	-	**-**	4.2
	2,3-Dimethyl-3-buten-2-ol	894	894	**-**	-	0.5	-	**-**	0.2
	Cyclohexanone	903	903	**-**	-	17.8	-	**-**	10.1
	Heptanal	906	905	1.0	2.1	-	1.1	0.8	-
	2-Butoxy ethanol	907	911	-	-	0.1	-	**-**	-
	1-Methylethyl benzene	929	930	-	-	0.4	-	**-**	0.2
	*α*-Pinene	930	929	-	-	-	1.0	**-**	-
	3-Ethyl-2-methyl heptane	942	941	-	-	-	-	**-**	0.1
	Camphene	953	954	-	-	-	0.5	**-**	-
	(*E)*-2-Heptenal	959	957	-	-	-	0.2	**-**	-
	Propyl benzene	960	960	-	-	4.6	-	**-**	3.0
	Benzaldeyhde	960	965	11.3	15.9	-	2.0	56.0	-
	1-Ethyl-3-methylbenzene	965	965	-	-	26.0	-	**-**	19.9
	1-Ethyl-4-methylbenzene	970	970	-	-	-	-	**-**	6.8
	Mesitylene	974	974	-	-	9.7	-	**-**	4.7
	6-Methyl-5-hepten-2-one	981	978	-	-	-	-	1.8	-
	Hydroxy benzene	980	981	-	3.0	-	-	**-**	-
	*psi*-Cumene	985	985	-	-	7.2	-	**-**	-
	1,2,4-Trimethylbenzene	985	987	-	-	-	-	-	32.2
	2-Pentyl furan	993	993	-	-	-	1.9	1.2	-
	Caprylaldeyhde	998	1002	0.8	2.4	-	-	1.4	-
	*δ*-3-carene	1001	1003	-	-	-	2.0	**-**	-
	(*Z*)-3-Hexenyl acetate	1004	1007	-	-	-	-	0.2	-
	Benzyl chloride	1019	1017	-	-	-	0.1	**-**	-
	*m*-Cymene	1027	1026	-	-	-	-	**-**	5.4
	Limonene	1031	1030	-	-	-	0.4	**-**	-
	Pentyl cyclohexane	1030	1032	-	-	0.1	-	**-**	0.1
	Hemellitol	1035	1036	-	-	7.5	-	**-**	-
	Benzene acetaldehyde	1036	1937	-	4.1	-	-	**-**	-
	Indane	1041	1040	-	-	1.2	-	**-**	1.0
	Salicylaldehyde	1045	1046	9.1	-	-	6.8	10.0	0.1
	1-Methyl-3-propylbenzene	1053	1053	-	-	1.6	-	**-**	1.4
	1,4-Diethylbenzene	1056	1056	-	-	-	-	**-**	0.4
	1-Ethyl-3,5-dimethylbenzene	1058	1059	-	-	1.1	-	**-**	-
	3-Methyldecane	1071	1067	-	-	0.3	-	**-**	0.3
	1-Methyl-2-propylbenzene	1074	1074	-	-	0.5	-	**-**	0.4
	4-Ethyl-1,2-dimethylbenzene	1077	1078	-	-	0.2	-	**-**	0.7
	1-Ethyl-2,4-dimethylbenzene	1083	1080	-	-	-	-	**-**	0.6
	2-Ethyl-1,4-dimethylbenzene	1085	1087	-	-	0.8	0.5	**-**	-
	Terpinolene	1086	1089	-	-	-		**-**	-
	Undecane	1100	1095	-	-	2.3	-	**-**	2.2
	Linalool	1095	1097	2.4	-	-	3.1	**-**	-
	Nonanal	1101	1101	20.5	31.6	0.1	4.5	3.3	-
	1-Ethyl-2,3-dimethylbenzene	1113	1109	-	-	0.2	-	-	0.2
	Phenylethyl alcohol	1120	1120	-	11.1	-	-	0.4	-
	1,5,8-*p*-Menthatriene	1128	1128	-	-	0.5	-	**-**	-
	Durene	1131	1132	-	-	0.8	-	**-**	0.6
	(2*E,*6*Z*)-Nonadienal	1150	1151	-	-	-	0.6	**-**	-
	(*E*)-2-Nonenal	1157	1157	0.2	-	-	0.5	**-**	-
	1,2,3,4-Tetramethyl benzene	1159	1155	-	-	-	-	**-**	0.3
	Naphthalene	1178	1179	-	7.0	-	-	2.1	0.4
	1-(3-Methylphenyl)ethanone	1171	1175	-	-	0.3	-	**-**	-
	*α*-Terpineol	1191	1192	-	-	-	0.6	**-**	-
	Methyl salicylate	1195	1197	0.7	0.6	-	5.0	0.6	0.3
	Decanal	1201	1201	0.8	2.0	-	2.0	1.5	-
	Dodecane	1200	1204	-	-	0.2	-	-	0.1
	*β*-Cylocitral	1224	1223	-	-	-	0.7	**-**	-
	(*E*)-2-Decanal	1260	1258	-	-	-	0.1	**-**	-
	Nonanoic acid	1267	1267	0.4	-	-	-	**-**	-
	Tridecane	1300	1303	1.0	-	-	-	**-**	0.1
	Undecanal	1305	1302	0.8	-	-	-	**-**	-
	Dehydro-ar-ionone	1355	1357	-	-	-	0.5	**-**	-
	(*E*)-*α*-Damascenone	1383	1386	0.3	-	-	-	**-**	-
	Tetradecane	1400	1402	0.7	-	0.4	-	**-**	0.6
	Lauric aldeyhde	1408	1406	0.7	1.0	-	-	**-**	-
	(*E*)-Caryophyllene	1417	1416	-	-	-	3.1	**-**	-
	Neryl acetone	1435	1439	-	-	-	0.9	**-**	-
	*α*-Humulene	1460	1460	-	-	-	0.4	**-**	-
	(*E*)-Ethyl cinnamate	1465	1468	-	4.9	-	-	2.0	-
	*α*-amorphene	1483	1481	-	-	-	0.7	**-**	-
	(*E)*-*β*-ionone	1487	1488	-	-	-	0.4	**-**	-
	Pentadecane	1500	1501	0.5	-	-	-	**-**	-
	Tridecylaldeyhde	1509	1505	1.3	-	-	-	**-**	-
	Tridecanal	1509	1507	-	1.3	-	-	**-**	-
	2,4-Di-tert-butylphenol	1502	1508	-	-	-	0.1	**-**	-
	*β*-Bisabolene	1509	1509	-	-	0.1	-	**-**	0.1
	*γ*-Cadinene	1513	1509	-	-	-	0.5	**-**	-
	Lauric acid	1565	1565	-	-	-	0.3	**-**	-
	Hexadecane	1600	1602	-	-	0.1	-	**-**	0.1
	Tetradecanal	1611	1608	1.2	0.6	-	-	**-**	-
	*α*-Bisabolol oxide B	1656	1660	-	-	-	0.4	**-**	-
	Heptadecane	1700	1701	-	-	-	1.8	1.8	0.1
	Pentadecanal	1710	1707	0.3	-	-	-	**-**	-
	Myristic acid	1763	1763	-	-	-	0.4	**-**	-
	Hexahydrofarnesyl acetone	1837	1838	-	-	-	1.3	**-**	-
	Hexadecanal	1811	1809	0.3	-	-	-	**-**	-
	Benzyl salicylate	1867	1865	0.8	-	-	0.9	**-**	-
	Nonadecane	1900	1899	0.7	-	-	-	**-**	-
	2-Heptadecanone	1901	1904	3.0	-	-	-	**-**	-
	(5*E*,9*E*)-Farnesyl acetone	1913	1915	-	-	-	0.2	**-**	-
	*n*-Hexadecanoic acid	1966	1965	0.2	-	-	0.8	**-**	-
	(*E,E*)-Geranyl linalool	2026	2025	0.1	-	-	-	**-**	-
	Heneicosane	2100	2099	4.4	-	-	0.6	**-**	-
	2-Nonadecanone	2101	2097	0.2	-	-	-	**-**	-
	Phytol	2110	2110	-	-	-	35.2	**-**	-
	Docosane	2200	2198	0.9	-	-	-	**-**	-
	Tricosane	2300	2300	29.6	2.0	-	4.0	**-**	-
	**Chemical classes**								
Monoterpene hydrocarbons				-	-	-	3.9	-	-
Oxygenated monoterpenes				2.4	-	-	4.8	-	-
Oxygenated diterpene				-	-	-	35.2	-	-
Sesquiterpene hydrocarbons				-	-	0.1	4.7	-	0.1
Aromatic hyd.				1.3	7	75	3.1	2.1	78.5
Aliphatic hyd.				41.0	2.0	3.7	6.4	1.8	3.8
Terpene related compounds				0.4	-	0.5	2.0	-	5.4
Aldehydes				49.5	68.8	0.1	22.1	86.6	0.1
Ketones				3.2	-	18.1	1.3	1.8	10.1
Esters				1.5	5.5	0.6	5.9	2.8	0.6
Others				0.6	14.1	1.8	8.8	4.7	1.2
Total				99.9	97.4	99.9	98.2	99.8	99.8

*Retention index of references; ^a^Retention index calculated from retention times relative to that of *n*-alkane (C_6_-C_32_) series. ^b ^Percentages obtained by

FID peak-area normalization; A1: EO of flower; A2: SPME of flower; A3: SPME of *n*-hexane extract of flower; B1: EO of stem-leaf; B2: SPME of

stem-leaf; B3: SPME of *n*-hexane extract of stem-leaf.

**Table 2:** Screening results for antimicrobial activity of the EOs, *n*-hexanes, methanol and aqueous extracts for the parts of *F. vulgaris*

**Samples of** *** F. vulgaris***		**Stoc. Sol. **(µg/mL)	**Microorganisms and inhibition zone (mm)**								
			***Ec***	***Yp***	***Pa***	***Sa***	***Ef***	***Bc***	***Ms***	***Ca***	***Sc***
Flower	EO	18.1	8	8	-	10	8	15	12	18	18
Stem-leaf	EO	13.4	8	-	-	6	-	6	18	6	10
Flower	HE	13.1	-	-	-	-	-	-	6	-	-
Stem-leaf	HE	10.2	-	-	-	-	-	-	7	-	-
Flower	ME	13.8	6	6	12	16	6	10	18	-	-
Stem-leaf	ME	16.6	-	6	10	13	-	8	8	-	-
Flower	AE	10.7	14	6	10	8	-	6	-	-	-
Stem-leaf	AE	10.9	-	-	-	6	-	-	-	-	-
Amp.		10	10	10	18	10	35	15			
Strep.		10							35		
Flu		5								25	25

Ec*: Escherichia coli*, Yp: *Yersinia pseudotuberculosis*, Pa: *Pseudomonas aeruginosa*, Sa: *Staphylococcus aureus*, *Enterococcus faecalis*,

Bc: *Bacillus cereus*, Ms: *Mycobacterium smegmatis*, Ca: *Candida albicans*, *Saccharomyces cerevisiae*. Amp.: Ampicillin, Str.: Streptomycin (-): Flu.:

Fluconazole, (-): No activity, HE: *n*-hexane extract; ME: methanol extract: AE: aqueous extract.

**Table 3.** Total phenolic content of methanol and aqueous extracts obtained from the flower and stem-leaf of *F. vulgaris*

**Samples of** *** F. vulgaris***		**Total phenolic ** **[** **mg gallic acid equivalents/g** **]**
Flower	ME	230.6 ± 12.1
Stem-leaf	ME	110.8 ± 8.6
Flower	AE	87.3 ± 5.6
Stem-leaf	AE	43. 4 ± 2.2

ME: methanol extract, AE: aqueous extract.

**Table 4:** Nitric oxide scavenging capacity of *n*-hexanes, methanol and aqueous extracts obtained from the flower and stem-leaf of *F. vulgaris*

**Samples of** *** F. vulgaris***		**IC**_50_ ** (μg/mL)**
Flower	HE	35.10 ± 3.02
Stem-leaf	HE	28.52 ± 2.41
Flower	ME	19.42 ± 2.21
Stem-leaf	ME	10.58 ± 1.66
Flower	AE	350.52 ± 5.85
Stem-leaf	AE	286.74 ± 6.23
*L*-ascorbic acid		5.55 ± 0.21

HE: *n*-hexane extract; ME: methanol extract: AE: aqueous extract.

**Table 5:** Tyrosinase inhibition potential of methanol and aqueous extracts obtained from the flower and stem-leaf of *F. vulgaris*

**Samples of** *** F. vulgaris***		**IC**_50_ ** (μg/mL)**
Flower	ME	0.58 ± 0.04
Stem-leaf	ME	0.28 ± 0.02
Flower	AE	6.05 ± 0.05
Stem-leaf	AE	4.62 ± 0.06
Kojic acid		3.12 ± 0.21

ME: methanol extract, AE: aqueous extract.
